# Localization of *tbg-1* mRNAs and GFP::TBG-1 protein in Early *C. elegans* Embryos​

**DOI:** 10.17912/W2CW8H

**Published:** 2017-08-23

**Authors:** Lauren DeMeyer, Mi Hye Song

**Affiliations:** 1 Department of Biological Sciences, Oakland University, Rochester, MI 48309

**Figure 1.**
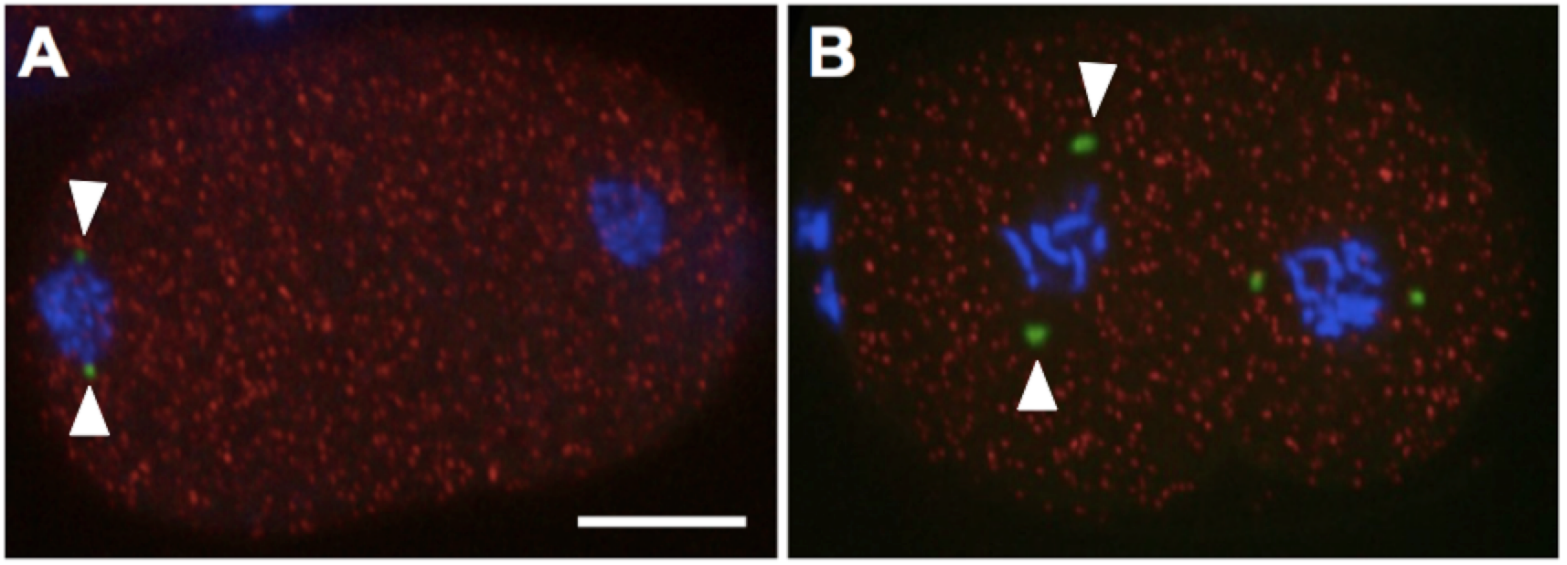


## Description

*tbg-1* encodes gamma-tubulin, a ubiquitous and highly conserved component of centrosomes in eukaryotic cells (Strome *et al*, 2001). Using smFISH we determined the localization of *tbg-1* transcripts (red). *tbg-1*transcripts are detected within distinct foci throughout the cytoplasm during both the first (A) and second (B) mitosis. *tbg-1* transcripts are not enriched at centrosomes or either blastomere. In contrast, GFP tagged TBG-1proteins (green signal; arrowheads) localize at centrosomes, as previously shown (Strome *et al*, 2001). Shown are projections from selected focal planes. Bar=10μm.

**​New Findings:** The first observation of *tbg-1* mRNA localization in early *C. elegans e*mbryos.

## Reagents

RNA probes targeting *tbg-1* mRNAs (Quasar 670; red) were designed using Stellaris Probe Designer (Biosearch Technologies). smFISH was performed as described previously (Osborne-Nishimura et al., 2015; Shaffer et al., 2013). For hybridization, embryos were incubated with *tbg-1* RNA probes (Quasar 670) at 39°C for four hours in the dark. Following hybridization, the embryos were washed and mounted with DAPI containing (blue) medium. To visualize centrosome-associated TBG-1 protein, we used transgenic strain that expresses GFP::TBG-1 (TH27; Hannak et al., (2002), green).
